# The ingenious ULKs: expanding the repertoire of the ULK complex with phosphoproteomics

**DOI:** 10.1080/15548627.2021.1968615

**Published:** 2021-09-14

**Authors:** Thomas J. Mercer, Sharon A. Tooze

**Affiliations:** The Francis Crick Institute, Molecular Cell Biology of Autophagy, London, UK

**Keywords:** AMPK, p150, PIK3R4, PRKAG2, VPS15, VPS34

## Abstract

The mammalian ULK kinase complex is the most upstream component in the macroautophagy/autophagy signaling pathway. ULK1 and homolog ULK2, the sole serine/threonine kinases in autophagy, transduce an array of autophagy-inducing stimuli to downstream autophagic machinery, regulating autophagy from autophagosome initiation to fusion of autophagosomes with lysosomes. ULK signaling is also implicated in a diverse array of non-canonical processes from necroptosis to ER-Golgi trafficking to stress granule clearance. However, the exact mechanisms by which ULK regulates these diverse processes remain largely unknown. Most notably, the number of validated ULK substrates is surprisingly low. Our study identifies new ULK substrates from a wide array of protein families and signaling pathways and supports an expanded range of physiological roles for the ULKs. We further characterize several new substrates, including the PIK3C3/VPS34-containing complex subunit PIK3R4/VPS15 and the AMPK component PRKAG2. Finally, by analyzing PIK3R4/VPS15-deficient models we discover novel aspects of ULK signaling with potential relevance in selective autophagy.

## Identifying novel ULK substrates

Using an array of mass spectrometry techniques to study the phosphoproteome of *ulk1^−/-^ ulk2^−/-^* double knockout cells, we generated high confidence lists of candidate substrates [1]. Direct *in vitro* phosphorylation was assayed leading to the identification of 20 novel ULK substrates (ACTG1, ANXA2, CARS, CHEK1, CTNND1, F11R, LAP3, NHSL1, PCM1, PRKAB2, PRKAG2, RALGPS2, SCEL, SORBS2, TBC1D1, UVRAG, VILL, VIM, PIK3R4/VPS15 and VPS26B), with the specific phosphoacceptor residue identified in the majority of cases. Furthermore, bioinformatics analysis provided insight into the range of ULK-dependent and -independent phosphoproteomic changes that occur when the kinases are removed. Our datasets give unprecedented insights into ULK signaling and provide a useful resource to advance our understanding of these substrates and their relevant pathways.

## New connections between autophagy signaling complexes

It is a fascinating question as to how a handful of kinase complexes facilitate the dramatic rearrangements of membranes and proteins required for autophagosome formation. The ULK complex plays an indispensable role in the process. It contains the regulatory components ATG13, ATG101 and dimeric RB1CC1/FIP200 along with either ULK1 or ULK2. While ULK1 and ULK2 share functions in autophagy, independent roles have been reported. Interestingly, by comparing the ability of ULK1 and ULK2 complexes to phosphorylate a diverse range of substrates in peptide array format, we showed that their substrate repertoires are virtually indistinguishable *in vitro*, indicating that any differences in roles between the paralogs are not due to varying target specificity.

The ULK complex coordinates nutrient and energy signals from the MTORC1 and AMPK complexes respectively. However, the interplay between ULK and AMPK signaling is controversial. ULK negatively regulates AMPK activity via an unknown mechanism, and AMPK has independently been shown to positively and negatively regulate ULK-dependent autophagy. We discovered that the nucleotide-sensing AMPK component PRKAG2, mutated in the cardiac glycogen storage disease PRKAG2 syndrome, is phosphorylated by both ULK and AMPK at serine 124, with phosphorylation sensitive to both serum and energy status. Based on previous PRKAG2 characterization studies, we speculate that phosphorylation might modulate AMPK complex localization. Our data therefore add an exciting new piece to the puzzle of ULK-AMPK regulation. Notably, as glycogen storage diseases are often associated with the impairment of autophagy, it is possible that further study of ULK-dependent PRKAG2 phosphorylation may yield disease-relevant insights.

Immediately downstream of the ULK complex is the class III phosphatidylinositol 3-kinase (PtdIns3K) PIK3C3/VPS34-containing complex. This is comprised of lipid kinase PIK3C3/VPS34 and regulatory subunits BECN1/Beclin1, PIK3R4/VPS15 and both NRBF2 and ATG14 (complex I), or UVRAG (complex II). Both generate the signaling phospholipid phosphatidylinositol-3-phosphate, but in different cellular contexts. Typically, complex I activity is associated with autophagy initiation and complex II activity with endolysosomal trafficking, as well as the later stages of autophagy.

### ULK: master regulator of the PtdIns3K complex

The regulation of PtdIns3K complex activity by ULK is an exciting and evolving area of study. ULK positively regulates PtdIns3K complex I upon autophagy initiation; however, a more comprehensive understanding of ULK-dependent regulation of both complexes is crucial. We identified PtdIns3K complex II component UVRAG as an *in vitro* and *in vivo* ULK substrate. Notably we also showed that VPS26B, part of the retromer complex which acts immediately downstream of PtdIns3K complex II in endolysosomal sorting, is phosphorylated by ULK at serine 302/304. We also present the first evidence of ULK-dependent PIK3C3/VPS34 serine 249 phosphorylation without kinase or substrate overexpression, and show that both nutrient and iron deprivation are upstream stimuli, giving physiological context to recent phenotypic data identifying serine 249 as a phosphorylation-activated LC3-interacting region.

**Figure 1.**
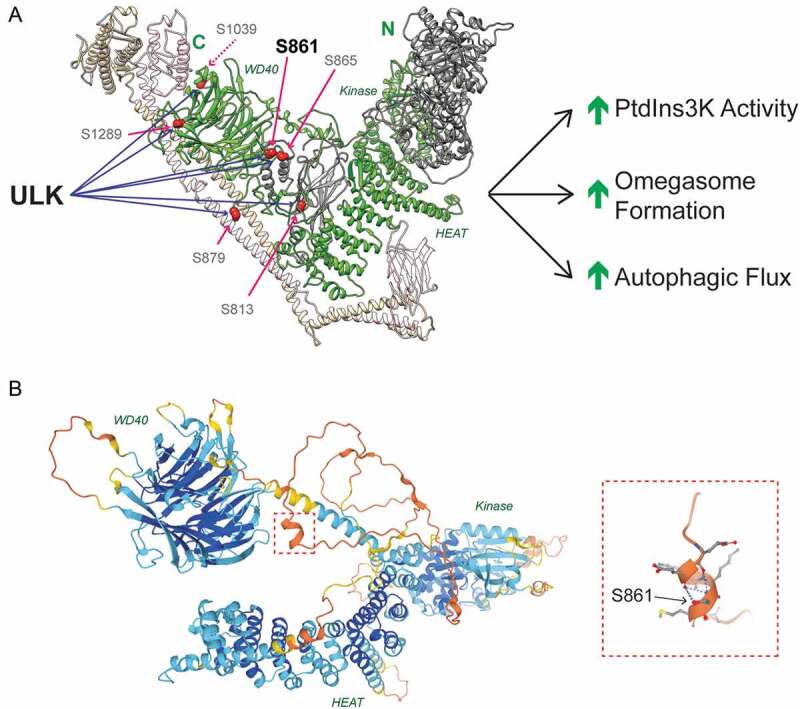
ULK regulates PtdIns3K activity by phosphorylating PIK3R4/VPS15. (A) Human PtdIns3K complex II with PIK3C3/VPS34 (gray), PIK3R4/VPS15 (green), BECN1 (beige) and UVRAG (pink).. PIK3R4/VPS15 domains are labeled in green. ULK phosphorylates PIK3R4/VPS15 at 6 sites, the major phosphoacceptor is serine 861. Phosphorylation promotes lipid kinase activity, omegasome formation and autophagic flux. (B) PIK3R4/VPS15 structural prediction from AlphaFold Protein Structural Database (see https://alphafold.ebi.ac.uk/entry/Q99570). Color indicates model confidence (navy = very high; aqua = confident; yellow = low; orange = very low). Serine 861 lies in the unstructured linker region connecting the HEAT and WD40 domains. The red dashed box indicates the region surrounding serine 861, which is magnified on the left hand side. Blue dashed lines indicate hydrogen bonds stabilizing the alpha helix

Importantly, we identified the pseudokinase PIK3R4/VPS15, as a novel ULK substrate. Previously thought to be an inert scaffold, recent structural insights indicate that PIK3R4/VPS15 may dynamically transmit activating signals to PIK3C3/VPS34’s lipid kinase domain. However, no functional post-translational modifications have been identified in PIK3R4/VPS15 to date. We identified 6 novel ULK substrate residues in PIK3R4/VPS15 (Figure 1A), mutation of which greatly reduced PIK3C3/VPS34 activity in vitro and omegasome formation in vivo. We showed that serine 861 is the major ULK phosphoacceptor in PIK3R4/VPS15 and is largely responsible for the cellular phenotypes. Together these data suggest that ULK-dependent PIK3R4/VPS15 phosphorylation promotes PIK3C3/VPS34 activity and autophagosome formation (Figure 1A). We identified the disordered linker between PIK3R4/VPS15’s HEAT and WD40 domains as a site of active and functional phospho-regulation. Intruigingly, structural predictions from the AlphaFold Protein Structure Database reveal that serine 861, within this linker, and its surrounding residues form a small alpha helix (Figure 1B). As serine 861 is predicted to stablize the helix, phosphorylation would likely modulate the local secondary structure, which we speculate may alter complex flexibility or accessory protein binding. Together, our data reposition PIK3R4/VPS15 as a driver rather than a passenger in PtdIns3K complex activation.

## ULK controls the distribution of autophagy proteins on cargo

To study the ULK-PIK3R4/VPS15 signaling axis, we generated the first human *PIK3R4/VPS15* knockout model. Unexpectedly, we discovered novel ULK-dependent phenotypes with potential relevance for the regulation of autophagy. We showed that upon PIK3R4/VPS15 ablation ULK phospho-substrates accumulate along with aberrant bodies positive for a range of autophagy proteins in PtdIns3K complex I-dependent manner. Our data revise previous findings identifying these bodies as stalled autolysosomes, instead favoring SQSTM1/p62-ubiquitin-positive aggregates, which sequester autophagic signaling proteins upon chronic inhibition of autophagic flux. The study of ubiquitin-induced SQSTM1 aggregation and phase separation has provided valuable insight into the mechanisms of redox homeostasis and selective autophagy. Our data suggest that these aggregates are sites of high local ULK activity, which actively remodels their outer surface by controlling the recruitment and distribution of autophagic machinery. Together, these data support a cargo-centric model for the initiation of selective autophagy, indicating that autophagy initiation machinery are subject to tight spatial regulation on cargo mediated by ULK kinase activity. Alongside recent data showing that multiple ULK complex translocations to distinct surface regions are required for the capture of mitochondria by autophagosomes, these data support the importance of spatial regulation of autophagic machinery in selective autophagy.
